# Efficacy and safety of ultrasound-guided compared to x-ray-guided percutaneous endoscopic lumbar discectomy in China: a systematic review and pooled analysis

**DOI:** 10.3389/fsurg.2025.1572977

**Published:** 2025-05-22

**Authors:** Bin Zheng, Panfeng Yu, Yan Liang, Zhenqi Zhu, Haiying Liu

**Affiliations:** Spine Surgery Department, Peking University People’s Hospital, Beijing, China

**Keywords:** ultrasound, x-ray, radiation, percutaneous endoscopic lumbar discectomy, meta

## Abstract

**Background:**

Percutaneous endoscopic lumbar discectomy (PELD) has become the preferred minimally invasive surgical treatment for lumbar disc herniation. This study aims to conduct a systematic literature review and meta-analysis to assess the efficacy and safety of ultrasound-guided PELD compared to x-ray-guided PELD.

**Methods:**

A comprehensive literature search was conducted in the PubMed, Cochrane Library, Ovid:MEDLINE, Embase, and China National Knowledge Infrastructure databases up to August 2024. Studies were included if they compared ultrasound- and x-ray-guided PELD in patients with lumbar disc herniation. Risk of bias and quality of evidence were assessed using the Cochrane Collaboration tools and the Newcastle–Ottawa Scale. The meta-analysis was performed using RevMan 5.4.

**Results:**

Seven studies were included, for a total of 767 patients (383 who underwent ultrasound-guided PELD and 384 who underwent x-ray-guided PELD). Ultrasound guidance significantly reduced fluoroscopy shots, radiation dose, fluoroscopy time, and working channel establishment time compared to x-ray guidance. Ultrasound guidance also demonstrated higher one-time puncture success rates. No significant differences were found in overall operative time, complications, postoperative pain scores (visual analog scale), or long-term functional outcomes (oxygen desaturation index and satisfaction rates).

**Conclusions:**

Ultrasound-guided PELD significantly reduces radiation exposure and improves puncture efficiency compared to x-ray-guided techniques while maintaining equivalent clinical outcomes and complication rates. However, due to study limitations, including small sample sizes and geographical concentration of research, further multicenter randomized controlled trials are necessary to validate these findings across diverse populations and surgical settings.

## Introduction

Percutaneous endoscopic lumbar discectomy (PELD) has gradually become the mainstream technique for the minimally invasive treatment of lumbar disc herniation due to its significant advantages over traditional surgery in terms of invasiveness, safety, precision, and complication rates (1–3). However, PELD has a steep learning curve, requiring proficient percutaneous puncture skills and a good understanding of spatial orientation. In clinical practice, percutaneous puncture is performed under fluoroscopic guidance using a C-arm x-ray, and this method often involves trial and error, as it heavily relies on the surgeon's experience. Repeated punctures increase radiation exposure for both the surgeon and the patient, especially for surgeons in the early stages of the learning curve (4–6). Therefore, the challenges associated with percutaneous puncture and related radiation exposure in PELD have drawn increasing attention from researchers (7, 8).

With the continuous improvement of ultrasound equipment and interventional ultrasound techniques, the application of ultrasound in areas such as the spine, including erector spinae plane blocks, is becoming more prevalent (9, 10). Ultrasound imaging does not cause radiation damage, occurs in real-time, and allows for visualization during surgery. Ultrasound imaging can dynamically generate real-time images without posing radiation risks to either the operator or the patient. Therefore, ultrasound guidance has practical significance in spine surgery. There remains a lack of systematic reviews or meta-analyses comparing ultrasound and X-ray guidance in PELD.

Herein, we aimed to conduct a comprehensive systematic literature review and meta-analysis to estimate the meta-analysis effect sizes and compare the efficacy and safety of ultrasound application in percutaneous endoscopic lumbar discectomy.

## Methods

### Study selection

A systematic review of the English literature available from the PubMed, Cochrane Library, Ovid:MEDLINE, and Embase databases was performed, along with a review of Chinese literature available from the China National Knowledge Infrastructure database from its inception to August 2024. The query utilized in the search was designed to include as many studies as possible pertaining to the outcomes of interest. The final search string was as follows: ((“percutaneous endoscopic lumbar discectomy” OR “PELD” OR “percutaneous transforaminal endoscopic discectomy” OR “PTED” OR “endoscopic spine surgery” OR “minimally invasive spine surgery”) AND (“ultrasound” OR “ultrasonography” OR “sonography” OR “ultrasonic” OR “US-guided” OR “ultrasound-guided”)). This study was performed according to a version of the Preferred Reporting Items for Systematic Reviews and Meta-Analyses (PRISMA) statement.

### Inclusion and exclusion criteria

Articles were included according to the following criteria:

(1) Patients: patients diagnosed with lumbar disc herniation who underwent PELD.
(2) Intervention: ultrasound-guided PELD surgery.
(3) Comparison: x-ray-guided PELD surgery.
(4) Outcomes: The study contains at least one of the following outcomes: Number of fluoroscopy shots, effective radiation dose, fluoroscopy time, one-time puncture success rates, working channel establishment time, operative time, complications, visual analog scale (VAS) score at follow-up, oxygen desaturation index (ODI) at follow-up, and satisfaction rate according to the Macnab criteria at follow-up.

Exclusion criteria:

(1) Patients with multi-segmental lumbar disc herniation, cauda equina syndrome, malignancy, or spinal deformity were excluded.
(2) Animal or cadaver experiment.
(3) No included outcomes.
(4) Conference abstracts, preprints, and dissertations were excluded.

### Study selection and data extraction

Two authors independently screened the literature by reading the titles, abstracts, and full texts and applying the inclusion and exclusion criteria. Author Panfeng Yu supervised the entire process and resolved all discrepancies.

Two researchers independently extracted the data and entered them into statistical software for statistical analysis. The data extraction included the following characteristics of the included studies: first author, publication year, study design, sample size, and outcomes.

### Evaluation of risk of bias

Only randomized controlled trials (RCTs) were eligible for the risk-of-bias assessment. Following the PRISMA and Cochrane Collaboration criteria, two authors independently assessed the risk of bias in the included studies in the following areas: (1) random sequence generation, (2) allocation concealment, (3) blinding of participants and personnel, (4) blinding of outcome assessment, (5) incomplete outcome data, (6) selective reporting, and (7) other bias. The Newcastle–Ottawa Scale was applied to evaluate the observational studies. If there was a disagreement, a decision was made through mutual consultation.

### Statistical analysis

The acquired data were analyzed using RevMan 5.4 software. Continuous outcomes were analyzed using the standard mean difference (SMD) and 95% confidence interval (CI). The odds ratio (OR) and 95% CI were used for dichotomous outcomes. A *P*-value <0.05 was considered to be statistically significant. Heterogeneity of the included literature was evaluated using the Q test (*χ*^2^) and I^2^. If the *P*-value was >0.05 and I^2^ <50%, it was considered that there was no significant statistical heterogeneity among the different studies, and a fixed effects model was used. If the *P*-value was <0.05 and I^2^ >50%, statistical heterogeneity existed, and a random-effects model was used.

## Results

### Study selection

**Figure 1:**
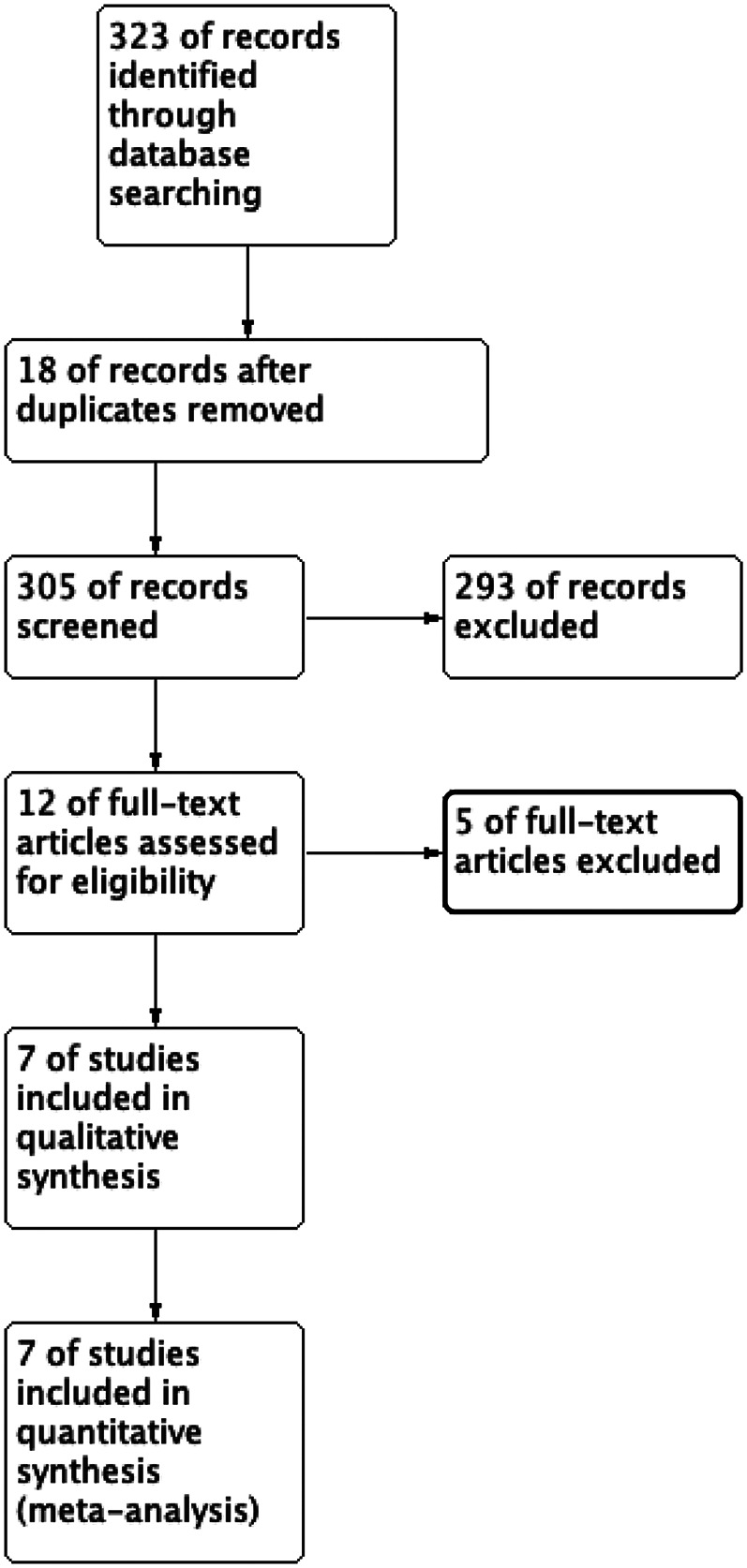
Study selection flow diagram.

The initial search included 323 studies, with the distribution as follows: PubMed 248, Embase 17, Ovid:MEDLINE 20, Cochrane 2, and CNKI 36 (accounting for 11.1% of the studies overall in the initial search). After removing duplicates, 305 articles were screened by title and abstract. After selection, seven studies met the inclusion criteria for data analysis. The study selection flowchart is shown in Figure 1. The seven eligible articles included seven comparison groups, with a combined 383 patients who underwent ultrasound-assisted PELD and 384 who underwent x-ray-assisted PELD (11–17). Of the seven included articles, four were found to be RCTs (11, 12, 15, 16), one was a cohort study (14), and two were case–control studies (13, 17). In total, 767 patients were included in this meta-analysis. The characteristics of the included studies are shown in Table 1.

**Table 1:** Characteristics of the included studies.

Study	Country	Study design	Sample size		BMI		Age		Follow-up	
			US	X-ray	US	X-ray	US	X-ray	US (months)	x-ray (months)
Qiu and Pan (12)	China	RCT	62	62	—	—	40.2 ± 3.8	39.7 ± 4.2	6	6
Wu et al. (14)	China	Cohort study	25	19	21.3 ± 1.8	21.5 ± 1.6	—	—	12	12
Sun (13)	China	Case–control	46	44	—	—	50 ± 6	48 ± 6	3	3
Wu et al. (15)	China	RCT	25	19	21.7 ± 1.8	21.4 ± 2.0	44.7 ± 16.1	42.9 ± 13.2	12	12
Zhu and Lin (17)	China	Case–control	42	51	—	—	43.7 ± 4.6	45.1 ± 3.8	12	12
Zhang et al. (16)	China	RCT	30	30	25.8 ± 3.5	25.9 ± 5.5	49.9 ± 20.1	42.8 ± 13.8	3	3
Li and Shen (11)	China	RCT	148	148	—	—	46.99 ± 3.78	47.01 ± 3.79	1	1

BMI = Weight (kg)/[height (m)*height (m)].

BMI, body mass index; RCT, randomized controlled trial; US, ultrasound.

### Risk of bias

**Figure 2:**
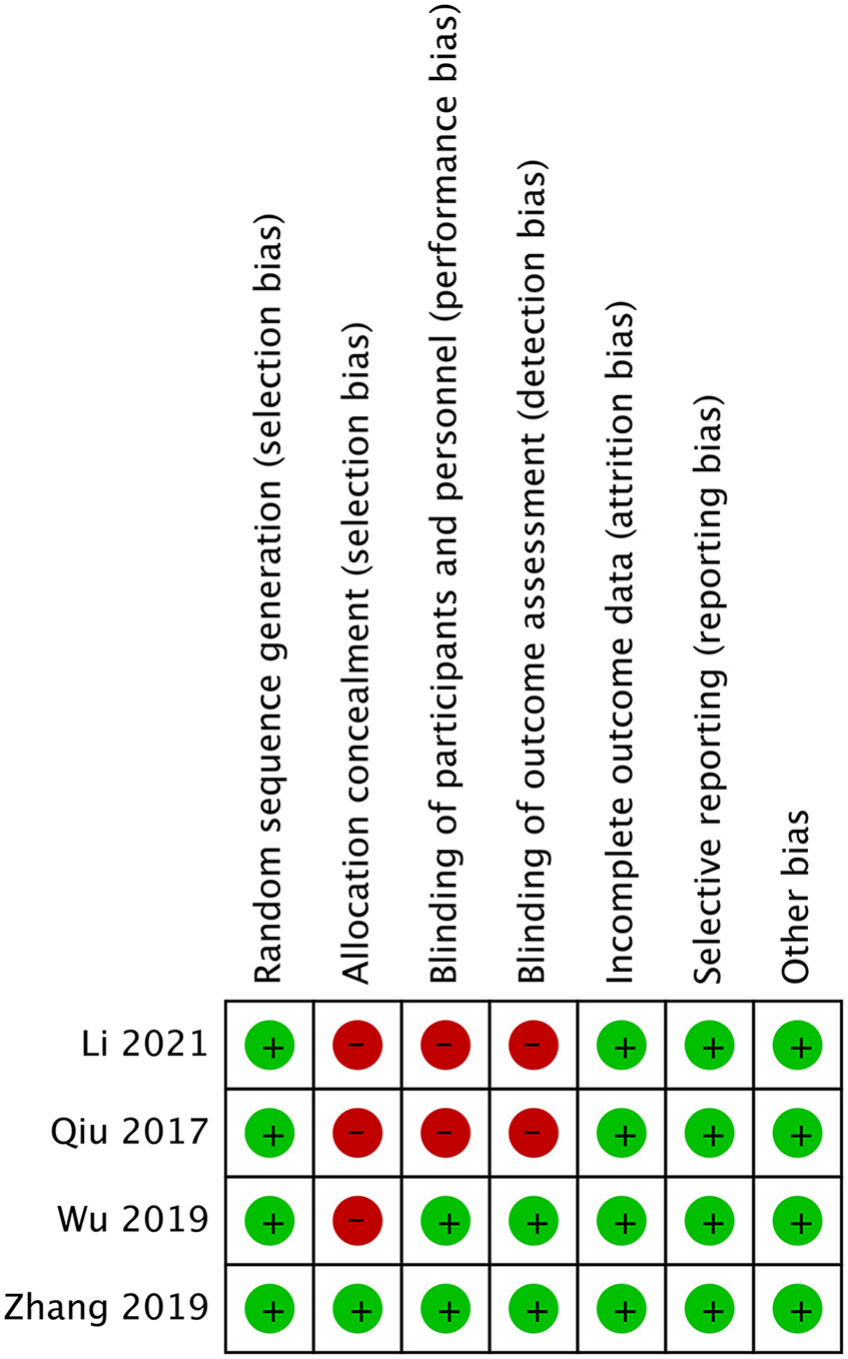
Risk of bias of randomized controlled trials.

We used the Cochrane Collaboration tool to evaluate the quality of the four RCTs. Details regarding the risk of bias in the two studies are shown in Figure 2. Zhang et al. (16) demonstrated low risk across all seven domains, representing the highest methodological quality. Wu et al. (15) showed high risk only in allocation concealment, while Li and Shen (11) and Qiu and Pan (12) exhibited high risk in three areas: allocation concealment, blinding of participants, and blinding of outcome assessment. These methodological limitations, particularly regarding blinding and allocation concealment, may have impacted the reliability and validity of findings in some of the included studies. Table 2 presents the Newcastle–Ottawa Scale quality assessment of the three observational studies on ultrasound-guided vs. x-ray-guided PELD. All the studies scored well in the selection and comparability domains (4/4 and 2/2 stars, respectively) but showed variations in the outcome domain. Zhu and Lin (17) demonstrated the highest methodological quality (eight stars), followed by Sun (13) (seven stars) and Wu et al. (14) (six stars). The primary quality differences are related to follow-up duration and completeness of outcome reporting.

**Table 2:** Risk of bias assessment using the Newcastle–Ottawa Scale for observational studies.

Study	Selection				Comparability		Outcome			Total scores
	Exposed cohort	Non-exposed cohort	Ascertainment of exposure	Outcome of interest	The most important factor	Additional factor	Assessment of outcomes	Length of follow-up	Adequacy of follow-up	
Wu et al. (14)	★	★	★	★	★	★				6
Sun (13)	★	★	★	★	★	★			★	7
Zhu and Lin (17)	★	★	★	★	★	★		★	★	8

### Fluoroscopy shots

**Figure 3:**
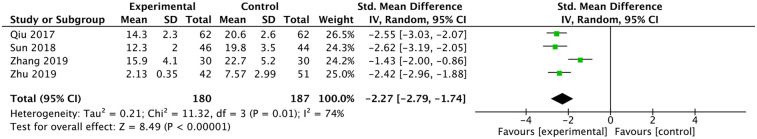
Comparison in fluoroscopy shots.

Four studies reported a comparison of the number of fluoroscopy shots (12, 13, 16, 17). They all reported fewer fluoroscopy shots in the ultrasound group and the meta-analysis had the same result [SMD: −2.27, 95% CI: (−2.79 to −1.74), *P* < 0.00001; heterogeneity Chi^2^ = 11.32, df = 3, *P* = 0.01, I^2^ = 74%], as shown in Figure 3.

### Effective radiation dose

**Figure 4:**
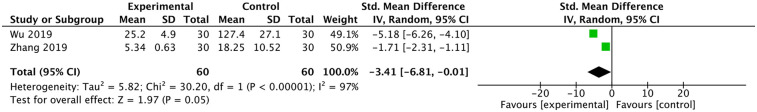
Comparison in radiation dose.

Two studies reported a comparison of radiation dose (15, 16). Both studies reported fewer shots in the ultrasound group, and the meta-analysis had the same result [SMD: −3.41, 95% CI: (−6.81 to −0.01), *P* = 0.05; heterogeneity Chi^2^ = 30.2, df = 1, *P* < 0.00001, I^2^ = 97%], as shown in Figure 4.

### Fluoroscopy time

**Figure 5:**
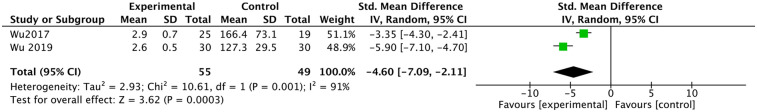
Comparison in fluoroscopy time.

Two studies reported a comparison of fluoroscopy time (14, 15). They all reported less fluoroscopy time in the ultrasound group, and the meta-analysis had the same result [SMD: −4.60, 95% CI: (−7.09 to −2.11), *P* = 0.0003; heterogeneity Chi^2^ = 10.61, df = 1, *P* =0.001, I^2^ = 91%], as shown in Figure 5.

### One-time puncture success rates

**Figure 6:**
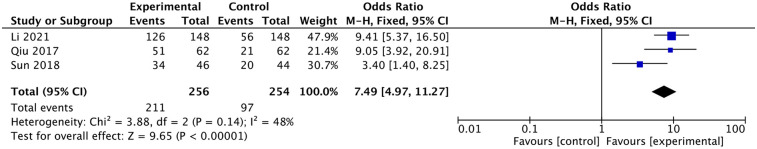
Comparison in onetime puncture success rate.

Three studies reported a comparison of the one-time puncture success rate (11–13). They all reported a higher one-time puncture success rate in the ultrasound group, and the meta-analysis had the same result [OR: 7.49, 95% CI: (4.97–11.27), *P* <0.00001; heterogeneity Chi^2^ = 3.88, df = 2, *P* = 0.14, I^2^ = 48%], as shown in Figure 6.

### Working channel establishment time

**Figure 7:**
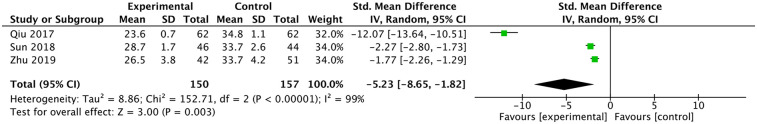
Comparison in working channel establishment time.

Three studies reported a comparison of working channel establishment time (12, 13, 17). They all reported a shorter working channel establishment time in the ultrasound group and the meta-analysis had the same result [SMD: −5.23, 95% CI: (−8.65, −1.82), *P* = 0.0003; heterogeneity Chi^2^ = 152.71, df = 2, *P* <0.00001, I^2^ = 99%] (Figure 7).

### Operative time

**Figure 8:**
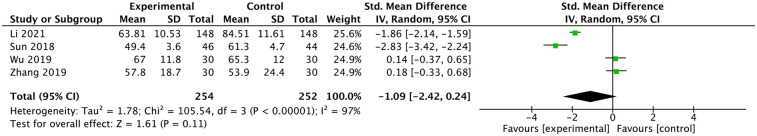
Comparison in operative time.

Four studies reported a comparison of operative times. Li and Shen (11) and Sun (13) reported shorter operative times in the ultrasound group (11, 13, 15, 16). Wu et al. (15) and Zhang et al. (16) reported similar operative times between the two groups. The meta-analysis showed no difference in operative times between the two groups [SMD: −1.09, 95% CI: (−2.42 to 0.24), *P* = 0.11; heterogeneity Chi^2^ = 105.54, df = 3, *P* <0.00001, I^2^ = 97%], as shown in Figure 8.

### Complications

**Figure 9:**
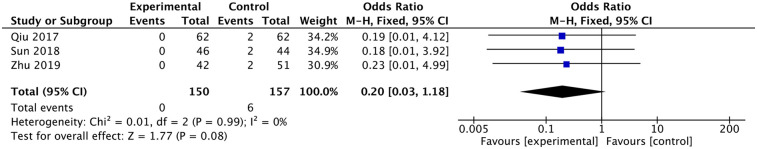
Comparison in complications.

Three studies reported a comparison of complications (12, 13, 17). Qiu and Pan (12) reported one case of intraoperative bleeding and one case of nerve root edema in the x-ray-guided PELD group. Sun (13) reported two cases of nerve root injury in the x-ray-guided PELD group, and Zhu and Lin (17) reported two cases of nerve root edema in the x-ray-guided PELD group. Patients who developed nerve root edema improved following postoperative symptomatic treatment. The meta-analysis showed no difference in complications between the two groups [OR: 0.2, 95% CI: (0.03–1.18), *P* =0.08; heterogeneity Chi^2^ = 0.01, df = 2, *P* = 0.99, I^2^ = 0%], as shown in Figure 9.

### VAS at follow-up

**Figure 10:**
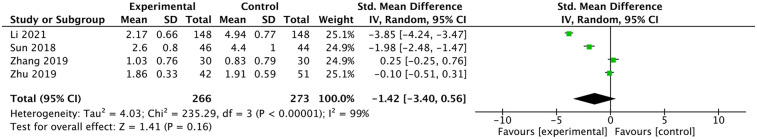
Comparison in VAS.

Four studies reported a comparison of VAS (11, 13, 16, 17). Li and Shen (11) and Sun (13) reported lower VAS scores in the ultrasound group. Zhu and Lin (17) and Zhang et al. (16) reported similar VAS scores between the two groups. The meta-analysis showed no difference in VAS scores between the two groups [SMD: −1.42, 95% CI: (−3.4 to 0.56), *P* =0.16; heterogeneity Chi^2^ = 235.29, df = 3, *P* <0.00001, I^2^ = 99%], as shown in Figure 10.

### ODI at follow-up

**Figure 11:**
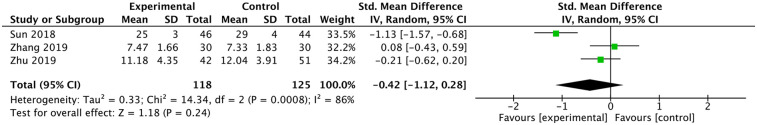
Comparison in ODI.

Sun (13) reported lower ODI in the Ultrasound-guided group. Zhang et al. (16) and Zhu and Lin (17) reported no difference in ODI. The meta-analysis indicated that there was no difference in ODI between the two groups [SMD: −0.42, 95% CI: (−1.12 to 0.28), *P* =0.24; heterogeneity Chi^2^ = 14.34, df = 2, *P* =0.0008, I^2^ = 86%], as shown in Figure 11.

### Satisfaction rate according to the Macnab criteria at follow-up

**Figure 12:**
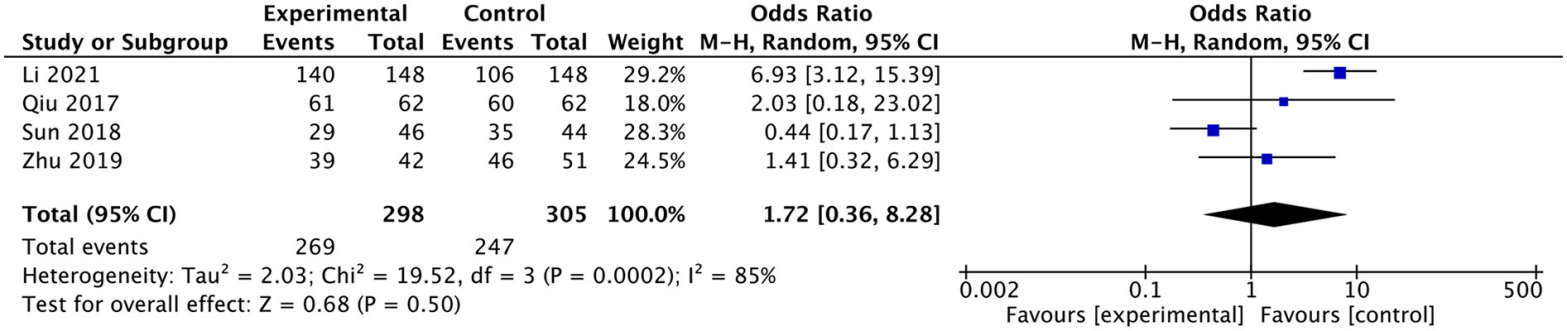
Comparison in satisfactory rate.

Li and Shen (11) reported higher satisfaction in the ultrasound group, but the other three studies reported no difference between the x-ray and ultrasound groups (11–13, 17). The meta-analysis indicated that there was no difference in satisfaction between the two groups [OR: 1.72, 95% CI: (0.36–8.28), *P* =0.5; heterogeneity Chi^2^ = 19.52, df = 3, *P* = 0.0002, I^2^ = 85%], as shown in Figure 12.

## Discussion

PELD has become a common surgical approach for treating lumbar disc herniation due to its minimally invasive nature, reliable efficacy, and fast postoperative recovery. However, PELD presents a steep learning curve, particularly for younger surgeons, as they face challenges with puncture skills and establishing the surgical pathway. Issues such as puncture failure, repeated punctures, and radiation exposure are problems that need to be addressed urgently (18–20). In recent years, ultrasound has been gradually applied in spinal surgery, where it has provided valuable assistance. Applications such as ultrasound-guided erector spinae plane block (21, 22) and spinal ultrasound for scoliosis screening underscore its potential in spinal surgery (23–25). Therefore, the use of ultrasound in spinal endoscopic surgery has promising prospects. However, there is still a lack of systematic reviews in this field. This study aimed to summarize existing research and conduct a meta-analysis to evaluate the role of ultrasound in endoscopic spine surgery.

Ultrasound imaging allows for real-time visualization of key anatomical landmarks, including the transverse processes, facet joints, ligamentum flavum, and interlaminar space. These structures appear as hyperechoic lines with distinct acoustic shadowing on ultrasound (11, 26). Specifically, the transverse process appears as a curved, hyperechoic structure with posterior acoustic shadowing, while the facet joint is visualized as a hyperechoic line with a small gap representing the articular space. This multi-dimensional view of spinal anatomy enables surgeons to plan an optimal puncture trajectory while avoiding neural structures and blood vessels. With ultrasound guidance, the puncture needle can be visualized in real-time using either an in-plane or out-of-plane approach. The in-plane technique allows visualization of the entire needle path, while the out-of-plane approach shows the needle tip as a hyperechoic dot (27). This continuous visualization eliminates the need for repeated fluoroscopic confirmation during the initial puncture phase. Fluoroscopy is then used minimally only to confirm the final working channel position before endoscopic insertion. In contrast, x-ray guidance only provides two-dimensional images, which require mental reconstruction of three-dimensional anatomy by the surgeon. This limitation often necessitates multiple fluoroscopic shots from different angles (anteroposterior and lateral views) to confirm needle positioning, resulting in increased radiation exposure and prolonged procedural time. The trial-and-error approach commonly used with x-ray guidance—where the needle is inserted, an image is taken, adjustments are made, and another image is taken—significantly increases the number of fluoroscopy shots and radiation dose.

In terms of radiological parameters, including radiation dose, fluoroscopy time, and number of fluoroscopy shots, all three outcome measures indicated that ultrasound-guided PELD results in significantly lower radiation exposure. The primary role of ultrasound in PELD is to assist with puncture and the establishment of the working channel, which are critical for a successful surgery and particularly challenging for young surgeons (28). Currently, puncture and channel placement largely rely on the surgeon's experience and are confirmed through repeated fluoroscopic imaging, which is the main source of radiation exposure. For patients, repeated fluoroscopy not only increases radiation exposure but also prolongs the duration of puncture and working channel establishment. The significant reduction in fluoroscopy shots (SMD: −2.27) and radiation dose (SMD: −3.41) represents more than just a statistical difference. For surgeons, especially those with high surgical volumes, this reduction may substantially lower their cumulative radiation exposure throughout their careers. Previous studies have confirmed that spine surgeons receive higher radiation doses than surgeons in other surgical specialties, potentially leading to long-term health effects, including increased cancer risk and, particularly, thyroid and ocular complications. The reduction observed in our study could make a meaningful contribution to occupational safety standards for minimally invasive spinal surgery (7).

With ultrasound assistance, the time required for the puncture and channel establishment is reduced, and the success rate of first-attempt punctures is higher. The benefits of ultrasound guidance include the following: (1) real-time continuous monitoring that is safe, radiation-free, and highly reproducible, with excellent resolution for surrounding soft tissues; (2) real-time assistance in visualizing the position of the puncture needle or working cannula, thereby increasing the success rates of the puncture and cannula placement and reducing operative time; and (3) the ability to detect abnormal local anatomical changes in the spine. Thus, ultrasound offers distinct advantages in endoscopic spinal surgery (29, 30). Despite the significant reduction in working channel establishment time, the overall operative time showed no statistical difference. Working channel establishment is only the initial phase of the entire PELD procedure. The PELD operation can be conceptually divided into two main stages: (1) the working channel establishment phase (affected by the guidance method) and (2) the endoscopic operation phase (including nucleus pulposus removal and neural decompression). In PELD surgery, the actual nucleus pulposus removal and neural decompression typically occupy the majority of the total operation time, and these steps are not influenced by the guidance method. In addition, the included studies showed extremely high heterogeneity in total operative time (I^2^ = 97%), indicating substantial variability between the different studies that is potentially related to factors such as the operating surgeons’ experience levels and learning curves (31).

The meta-analysis showed that the difference in complications between the two groups did not reach statistical significance (*P* = 0.08). However, it is noteworthy that all of the reported complications occurred in the x-ray group, while the ultrasound group demonstrated a significantly higher first-attempt puncture success rate The complications reported in this study primarily centered on nerve root injuries, including nerve root edema and direct nerve root damage. In the x-ray-guided group, Qiu and Pan (12) reported one case of intraoperative bleeding and one case of nerve root edema; Sun (13) reported two cases of nerve root injury; and Zhu and Lin (17) reported two cases of nerve root edema. These complications may be associated with repeated puncture attempts and nerve root damage during foramen exit. X-ray-guided puncture typically employs a trial-and-error approach, requiring multiple needle position adjustments and repeated x-ray imaging to confirm placement, which increases the risk of repeated punctures and potentially leads to surrounding tissue damage, particularly to nerve roots. In contrast, ultrasound guidance provides real-time visualization of the puncture pathway, significantly improving the first-attempt puncture success rate, thereby potentially reducing the risk of nerve root injury. Although the meta-analysis showed no statistically significant difference in overall complications between the two groups, the more precise puncture and reduced operative times with ultrasound guidance may offer potential advantages in reducing nerve-related complications.

In the follow-up of postoperative outcomes, there was no difference in VAS, ODI, or satisfaction rates between the ultrasound and x-ray groups. The differences between x-ray-guided and ultrasound-guided PELD mainly occur during the puncture and channel establishment process. The postoperative clinical outcomes (such as VAS, ODI, and satisfaction) primarily depend on the effectiveness of decompression in the second stage, specifically whether the surgery adequately removed the protruding material and bony stenosis compressing the nerve roots. Since the patients in both groups received identical surgical procedures during this stage, performed by professional surgeons achieving the same decompression effect, the similar long-term clinical results were as expected (16).

Limitations: (1) The number of included studies was limited, with only seven studies incorporated into the meta-analysis, and the sample size was relatively small. Among these, only three were RCTs, and in terms of study quality, high-quality RCTs can provide a higher level of evidence. (2) The meta-analysis only included small, single-center studies, which limits the generalizability. Small sample sizes may lead to insufficient statistical power, increasing the risk of both false negative and false positive results. (3) All the included studies were from China, which may not fully reflect the differences in influencing factors, such as ethnicity, surgical habits, and equipment differences between countries. This could introduce regional bias and affect the generalizability of the findings. Future research should incorporate multinational data to validate the broader applicability of ultrasound-guided PELD. (4) This study only included publicly published literature in Chinese and English, excluding literature in other languages, which may introduce bias to the research conclusions. (5) For outcomes limited to only two studies, the small sample sizes made the results susceptible to random errors, and the heterogeneity assessments had limited power. For example, only two studies reported effective radiation dose, and three studies reported complications. This lack of data may have affected the reliability of the meta-analysis. Nevertheless, these preliminary findings remain valuable and can serve as a foundation for more comprehensive future meta-analyses, although the interpretation of the results requires caution. (6) The high heterogeneity (I^2^ = 97%–99%) in the key outcomes indicated substantial between-study variability. With only seven included studies and three to four studies per outcome, we could not conduct subgroup analyses or meta-regression to investigate the sources of the heterogeneity. We addressed these limitations by using random-effects models for outcomes with high heterogeneity and transparently reported all heterogeneity statistics to support the appropriate interpretation. Therefore, in the future, it is necessary to conduct multicenter, large-sample RCTs to further clarify the effectiveness and safety of ultrasound guidance in PELD.

## Conclusion

Ultrasound-guided PELD significantly reduces radiation exposure and improves puncture efficiency compared to x-ray-guided techniques while maintaining equivalent clinical outcomes and complication rates. However, study limitations, including small sample sizes and geographical concentration of research, necessitate further multicenter randomized controlled trials to validate these findings across diverse populations and surgical settings.

## Data Availability

The raw data supporting the conclusions of this article will be made available by the authors without undue reservation.

## References

[1] Gadjradj PS Harhangi BS Amelink J van Susante J Kamper S van Tulder M Percutaneous transforaminal endoscopic discectomy versus open microdiscectomy for lumbar disc herniation: a systematic review and meta-analysis. Spine (Phila Pa 1976). (2021) 46(8):538–49. 10.1097/BRS.0000000000003843 33290374 PMC7993912

[2] Li X Han Y Di Z Cui J Pan J Yang M Percutaneous endoscopic lumbar discectomy for lumbar disc herniation. J Clin Neurosci. (2016 Nov) 33:19–27. 10.1016/j.jocn.2016.01.043 27475315

[3] Pan M Li Q Li S Mao H Meng B Zhou F Percutaneous endoscopic lumbar discectomy: indications and complications. Pain Physician. (2020) 23(1):49–56. 32013278

[4] Ahn SS Kim SH Kim DW. Learning curve of percutaneous endoscopic lumbar discectomy based on the period (early vs. late) and technique (in-and-out vs. In-and-out-and-in): a retrospective comparative study. J Korean Neurosurg Soc. (2015) 58(6):539–46. 10.3340/jkns.2015.58.6.539 26819689 PMC4728092

[5] Lee DY Lee SH. Learning curve for percutaneous endoscopic lumbar discectomy. Neurol Med Chir (Tokyo). (2008) 48(9):383–8. discussion 8–9. 10.2176/nmc.48.383 18812679

[6] Sun B Shi C Xu Z Wu H Zhang Y Chen Y Learning curve for percutaneous endoscopic lumbar diskectomy in bi-needle technique using cumulative summation test for learning curve. World Neurosurg. (2019) 129:e586–e93. 10.1016/j.wneu.2019.05.227 31158541

[7] Ahn Y Kim CH Lee JH Lee SH Kim JS. Radiation exposure to the surgeon during percutaneous endoscopic lumbar discectomy: a prospective study. Spine (Phila Pa 1976). (2013) 38(7):617–25. 10.1097/BRS.0b013e318275ca58 23026867

[8] Fan G Gu X Liu Y Wu X Zhang H Gu G Lower learning difficulty and fluoroscopy reduction of transforaminal percutaneous endoscopic lumbar discectomy with an accurate preoperative location method. Pain Physician. (2016) 19(8):E1123. 27906942

[9] Ma J Bi Y Zhang Y Zhu Y Wu Y Ye Y Erector spinae plane block for postoperative analgesia in spine surgery: a systematic review and meta-analysis. Eur Spine J. (2021) 30(11):3137–49. 10.1007/s00586-021-06853-w 33983515

[10] Oh SK Lim BG Won YJ Lee DK Kim SS. Analgesic efficacy of erector spinae plane block in lumbar spine surgery: a systematic review and meta-analysis. J Clin Anesth. (2022) 78:110647. 10.1016/j.jclinane.2022.110647 35030493

[11] Li P Shen Z. Clinical effect of ultrasound-guided spinal endoscopy in patients with lumbar disc herniation. Med Innov China. (2021) 18(23):43–7.

[12] Qiu P Pan T. Clinical study of ultrasound-guided spinal endoscopy in the treatment of lumbar disc herniation. Chin J Pain Med. (2017) 23(11):861–4.

[13] Sun X. Clinical efficacy of ultrasound-guided spine endoscopy in the treatment of lumbar disc herniation. Chin Remedies Clin. (2018) 18(10):1786–8.

[14] Wu R Liao X Xia H. Radiation exposure to the surgeon during ultrasound-assisted transforaminal percutaneous endoscopic lumbar discectomy: a prospective study. World Neurosurg. (2017) 101:658–65.e1. 10.1016/j.wneu.2017.03.050 28342919

[15] Wu RH Deng DH Huang XQ Shi CL Liao XQ. Radiation exposure reduction in ultrasound-guided transforaminal percutaneous endoscopic lumbar discectomy for lumbar disc herniation: a randomized controlled trial. World Neurosurg. (2019) 124:e633–e40. 10.1016/j.wneu.2018.12.169 30648611

[16] Zhang M Yan L Li S Li Y Huang P. Ultrasound-guided transforaminal percutaneous endoscopic lumbar discectomy: a new guidance method that reduces radiation doses. Eur Spine J. (2019) 28(11):2543–50. 10.1007/s00586-019-05980-9 31087164

[17] Zhu L Lin H. Clinical value of ultrasound-guided interventional techniques in full-endoscopic treatment of lumbar disc herniation. J Cervicodynia Lumbodynia. 2019;40(06):857–8.

[18] Sun B Shi C Xu Z Wu H Zhang Y Chen Y Learning curve for percutaneous endoscopic lumbar diskectomy in bi-needle technique using cumulative summation test for learning curve. World Neurosurg. (2019) 129:e586–e93. 10.1016/j.wneu.2019.05.227 31158541

[19] Wang H Huang B Li C Zhang Z Wang J Zheng W Learning curve for percutaneous endoscopic lumbar discectomy depending on the surgeon’s Training level of minimally invasive spine surgery. Clin Neurol Neurosurg. (2013) 115(10):1987–91. 10.1016/j.clineuro.2013.06.008 23830496

[20] Wu X-b Fan G-x Gu X Shen T-g Guan X-f Hu A-n Learning curves of percutaneous endoscopic lumbar discectomy in transforaminal approach at the L4/5 and L5/S1 levels: a comparative study. J Zhejiang Univ Sci B. (2016) 17(7):553. 10.1631/jzus.B1600002 27381732 PMC4940631

[21] Peng Q Meng B Yang S Ban Z Zhang Y Hu M Efficacy and safety of erector spinae plane block versus thoracolumbar interfascial plane block in patients undergoing spine surgery: a systematic review and meta-analysis. Clin J Pain. (2024) 40(2):114–23. 37982694 10.1097/AJP.0000000000001177

[22] Wilson AA Schmid AM Pestaña P Tubog TD. Erector spinae plane block on postoperative pain and opioid consumption after lumbar spine surgery: a systematic review and meta-analysis of randomized controlled trials. J Perianesth Nurs. (2024) 39(1):122–31. 10.1016/j.jopan.2023.06.003 37747377

[23] de Reuver S Moens A Kruyt MC Nievelstein RAJ Ito K Castelein RM. Ultrasound shear wave elastography of the intervertebral disc and idiopathic scoliosis: a systematic review. Ultrasound Med Biol. (2022) 48(5):721–9. 10.1016/j.ultrasmedbio.2022.01.014 35232608

[24] Ungi T Greer H Sunderland KR Wu V Baum ZMC Schlenger C Automatic spine ultrasound segmentation for scoliosis visualization and measurement. IEEE Trans Biomed Eng. (2020) 67(11):3234–41. 10.1109/TBME.2020.2980540 32167884 PMC7654705

[25] Wu HD Liu W Wong MS. Reliability and validity of lateral curvature assessments using clinical ultrasound for the patients with scoliosis: a systematic review. Eur Spine J. (2020) 29(4):717–25. 10.1007/s00586-019-06280-y 31925562

[26] Barrington MJ Wong DM Slater B Ivanusic JJ Ovens M. Ultrasound-guided regional anesthesia: how much practice do novices require before achieving competency in ultrasound needle visualization using a cadaver model. Reg Anesth Pain Med. (2012) 37(3):334–9. 10.1097/AAP.0b013e3182475fba 22354107

[27] Bowness J Taylor A. Ultrasound-guided regional anaesthesia: visualising the nerve and needle. Biomed Vis. (2020) 6:19–34. 10.1007/978-3-030-37639-0_2 32488634

[28] Xiao X Chen L Hu X Wang Y Saini A Liu T Ultrasound robotic system to multi-approach puncture for endoscopic spinal surgery. IEEE Robot Autom Lett. (2024) 9(10):9119–26. 10.1109/LRA.2024.3440090

[29] Ahmed AS Ramakrishnan R Ramachandran V Ramachandran SS Phan K Antonsen EL. Ultrasound diagnosis and therapeutic intervention in the spine. J Spine Surg. (2018) 4(2):423. 10.21037/jss.2018.04.06 30069538 PMC6046321

[30] Darrieutort-Laffite C Hamel O Glemarec J Maugars Y Le Goff B. Ultrasonography of the lumbar spine: sonoanatomy and practical applications. Joint Bone Spine. (2014) 81(2):130–6. 10.1016/j.jbspin.2013.10.009 24618457

[31] Maayan O Pajak A Shahi P Asada T Subramanian T Araghi K Percutaneous transforaminal endoscopic discectomy learning curve: a CuSum analysis. Spine (Phila Pa 1976). (2023) 48(21):1508–16. 10.1097/BRS.0000000000004730 37235810

